# Utility of NICaS Non-Invasive Hemodynamic Monitoring in Critically Ill Patients with COVID-19

**DOI:** 10.3390/jcm13072072

**Published:** 2024-04-03

**Authors:** Wisam Zabeeda, Jonah Benjamin Cohen, Anat Reiner Benaim, Shiri Zarour, Yael Lichter, Idit Matot, Or Goren

**Affiliations:** 1 Department of Anesthesiology, Pain and Intensive Care, Tel Aviv Medical Center, Tel Aviv University, Tel Aviv 6997801, Israel; wisamz@tlvmc.gov.il (W.Z.); shiriph@tlvmc.gov.il (S.Z.); yaelli@tlvmc.gov.il (Y.L.); iditm@tlvmc.gov.il (I.M.); org@tlvmc.gov.il (O.G.); 2 Department of Epidemiology, Biostatistics and Community Health Sciences, School of Public Health, Faculty of Health Sciences, Ben Gurion University of the Negev, Beer Sheva 8410501, Israel; reinera@bgu.ac.il

**Keywords:** COVID-19, hemodynamic monitor, cardiac function

## Abstract

(1) **Background**: COVID-19 presented many challenges to effective treatments, such as managing cardiovascular insufficiency while mitigating risks to healthcare providers. This study utilized NICaS, a non-invasive hemodynamic monitor that provides advanced data via whole-body impedance analysis. We investigated the associated trends in hemodynamic parameters obtained by the NICaS device and their correlation with in-hospital all-cause mortality during COVID-19 hospitalization in the intensive care unit. (2) **Methods:** Data from 29 patients with COVID-19 admitted to the intensive care unit and monitored with NICaS between April 2020 and February 2021 were analyzed retrospectively. (3) **Results:** Decreasing cardiac output and cardiac power were significantly associated with death. Total peripheral resistance was significantly increasing in non-survivors as was total body water percentage. Those admitted with a heart rate above 90 beats per minute had a significantly reduced survival. (4) **Conclusions:** Non-invasive hemodynamic monitoring via the NICaS device is simple and effective in evaluating critically ill patients with COVID-19 and may help guide clinical management via remote monitoring. Controlling tachycardia may help ensure adequate oxygen supply-demand ratio. A hint toward a beneficiary effect of a restrictive fluid balance may be observed.

## 1. Introduction

The novel coronavirus (COVID-19) that hit the world in 2019 created unexpected obstacles to medical personnel’s effective and efficient responses. In addition to treating the respiratory complications of COVID-19, managing cardiovascular insufficiency was a significant concern. Cardiovascular insufficiency may be exacerbated by several factors, such as hypovolemia secondary to fever and dehydration or due to deliberate fluid restriction as part of the treatment of acute respiratory distress syndrome (ARDS) [1]; ventricular dysfunction due to myocarditis and the exacerbation of a pre-existing heart disease [2]; acute cor pulmonale secondary to positive pressure ventilation [3]; alterations in venous return due to a multisystem inflammatory process and positive pressure ventilation [4]; and hypercoagulability leading to pulmonary embolism and complications thereafter [5]. 

Another challenge in the management of COVID-19 concerns the high virulence of the pathogen. Since COVID-19 is highly contagious, treating patients requires appropriate personal protective equipment (PPE) that meets the contact, airborne, and droplet precautions [6,7]. Therefore, approaches were developed to reduce or simplify the direct examination and testing of patients that could otherwise increase healthcare workers’ exposure to the disease [8]. One such method is remote monitoring.

The NICaS (NI Medical, Ra’anana, Israel, Version 3.63.15) device is an FDA-approved, CE-marked, non-invasive impedance-based hemodynamic monitor [9]. Measurements can be performed in a simple, fast, non-invasive fashion by healthcare providers regardless of their level of training. Two stickers connected to the device are placed on two of the patient’s limbs. Data can be recorded, and trends can be observed over time. The hemodynamic parameters collected include the cardiac index (CI) in L/min/m^2^, stroke volume index (SI) in ml/m^2^, cardiac power index (CPI) in watts/m^2^, total peripheral resistance index (TPRI) in dyn*s*cm^−5^/m^2^, and total body water percentage (TBW). Respiratory rates and heart rates (HRs) are also measured. The utility and validity of NICaS as a tool for non-invasive hemodynamic monitoring were compared with gold-standard testing [10].

There are scarce data regarding advanced hemodynamic parameters during COVID-19 ICU hospitalization obtained through non-invasive means other than echocardiography. In addition, to the best of our knowledge, the utility of the NICaS device in COVID-19 ICU patients has not been assessed. The potential advantages of using a non-invasive hemodynamic monitor, which provides high-fidelity real-time measurements and minimizes direct contact with the patient and potential harm to medical personnel, deserve attention in a pandemic with limited resources.

Therefore, we aimed to investigate the associated trends in hemodynamic parameters obtained by the NICaS device and in-hospital all-cause mortality during COVID-19 ICU hospitalization. 

## 2. Methods

### 2.1. Study Design and Patient Population

We conducted a single-center retrospective cohort study at a tertiary referral center. The institutional review board approved this study, waiving the need for individual consent. We included all confirmed patients with COVID-19 admitted to the ICU who underwent non-invasive hemodynamic monitoring with NICaS (NI Medical, Ra’anana, Israel, Version 3.63.15) in the Tel-Aviv Medical Center (TLVMC, Tel Aviv, Israel) between April 2020 and February 2021. Pregnant patients and minors under 18 years old were excluded from this study.

### 2.2. Data Collection

During the pandemic, our hospital treated hundreds of patients, and the monitoring modality used was decided according to the treating physicians’ clinical discretion and according to personal familiarity and preferences. 

We were able to locate the patients monitored using NICaS through the computerized registry of the NICaS monitor. Altogether, 29 patients were evaluated using the NICaS monitor during the pandemic. 

Data were obtained from electronic medical record systems and the NICaS device for the entire length of the index hospital stay. Data included demographics; medical history, including chronic medication use; clinical variables regarding the ICU hospitalization period (including illness severity scores, hemodynamic data, laboratory results, medication administered, echocardiography test results, ICU length of stay); discharge destination; and in-hospital all-cause mortality. Hemodynamic data were extracted retrospectively from the NICaS device. According to ICU protocol during the pandemic, the ICU team evaluated the hemodynamic parameters (in all monitors used) in intervals of 24 to 72 h until ICU discharge or death.

In order to compare the study patients to the local COVID-19 population, we used the hospital’s database of critically ill patients. However, this database is more extensive since it includes patients who were admitted to ICUs and intermediate care units. On the other hand, all the patients monitored by NICaS were admitted to ICUs only. 

### 2.3. Study Outcomes

In our primary analysis, we investigated the association between trends in hemodynamic measurements during ICU hospitalization, as captured by the NICaS device, and in-hospital all-cause mortality. 

In the secondary analysis, we explored the association between initial hemodynamic measurements upon admission and overall survival.

In the pre-defined exploratory sub-group analysis, we aimed to assess possible systematic differences between the initial stroke volume index as measured by NICaS and echocardiography upon admission. 

### 2.4. Statistical Analyses

The categorical variables are presented as frequencies and percentages. The continuous variables are reported as medians and interquartile range (IQR). 

Univariate analysis of the categorical variables was conducted using the Fisher exact test and Mann–Whitney test when analyzing continuous variables. 

The multivariate analysis used a mixed-effects model with a backward elimination procedure to assess the association between trends in hemodynamic measurements and in-hospital all-cause mortality. 

The secondary analysis evaluated the association between the initial hemodynamic measurements and overall survival via the Kaplan–Meier method and the log-rank test. 

Bland–Altman analysis was used to test the level of agreement between these two measurement methods.

All statistical tests were two-sided; *p* < 0.05 was considered statistically significant. Statistical analysis was conducted using the SPSS software (IBM SPSS Statistics for Windows, version 27, IBM Corporation, Armonk, NY, USA, 2020) and R Statistical software (2023). R: A Language and Environment for Statistical Computing, R Foundation for Statistical Computing, Vienna, Austria). 

### 2.5. Sample Size–Power Calculation

Our study is a limited-size retrospective study. Therefore, we have not tested our study for power or sample size.

## 3. Results

### 3.1. Study Cohort

The study cohort included 29 patients monitored by NICaS during their stay in the ICU. The median [IQR] age was 66.00 [46.00, 79.00] years, and 8.00 (28.00%) were women. Other baseline characteristics, comorbidities, hemodynamic variables, and medical data regarding ICU admission are presented in Table 1.

When comparing the study patients to a database of 488 patients with COVID-19 admitted during the same period, it appears that the demographic parameters and comorbidities are similar between the study population and the general critically ill COVID-19 population (in the general population, the median age was 66.00 [IQR 51.00–77.00], *p* 0.99; median BMI was 26.83 [IQR 23.67–30.11], *p* 0.57; 187.00 were women (28.31%), *p* 0.25; 103.00 (21.10%) suffered from a cardiac disease, *p* 0.09; 81.00 (16.60%) suffered from respiratory disease, *p* 0.29; 229.00 (46.90%) were previously diagnosed with hypertension, *p* 0.22; 173.00 (35.50%) were previously diagnosed with hyperlipidemia, *p* 0.31; 53.00 (10.90%) had CKD, *p* 0.07; and 157.00 (32.20%) had DM, *p* 0.37). However, the larger cohort of 488 patients had better baseline conditions (median HR 86.00 [IQR 75.00–96.00], *p* < 0.01; median saturation 95.00 [IQR 90.00–98.00], *p* < 0.01; median Sequential Organ Failure Assessment score (SOFA) 1.00 [IQR 0.00–2.00], *p* 0.01; and median Modified Early Warning Score (MEWS) 3.00 [IQR 0.00–6.00], *p* < 0.01) than the study population. 

The median [IQR] number of measurement intervals obtained from the NICaS device in the study population was three [2,3,4]. The median CI was 2.48 [IQR 1.92–3.05], the median TPRI was 3105.67 [IQR 2190.88–3627.00], the median CPI was 0.44 [IQR 0.33–0.55], the median SI was 28.00 [IQR 22.41–35.21], and the median TBW was 49.15 [IQR 42.55, 54.65].

It is worth noting that the mean cardiac index was lower among CKD patients (1.95 ± 0.66 vs. 3.84 ± 5.80, *p* 0.02), while the mean TPRI was higher among diabetic patients (4106.12 ± 1068.89 vs. 2807.68 ± 1044.87, *p* 0.03) and among CKD patients (4092.89 ± 1152.48 vs. 2811.88 ± 1024.73, *p* < 0.01). In addition, the mean CPI was lower among CKD patients (0.36 ± 0.14 vs. 0.49 ± 0.16, *p* 0.05) and higher among patients receiving antiIL6 (0.52 ± 0.18 vs. 0.38 ± 0.10, *p* < 0.01).

The in-hospital all-cause mortality rate was 9.00 (31.00%). Of the patients who survived index admission, 12.00 (60.00%) patients were discharged home, and 8.00 (40.00%) patients were discharged to a rehabilitation facility. No significant differences in the hemodynamic parameters were found between patients discharged home and those discharged to rehabilitation facilities.

### 3.2. Primary Analysis

A linear multivariate mixed-effects analysis showed a statistically significant decline in CI during ICU hospitalization among non-survivors compared to stable levels among survivors (*p* 0.02); similarly, the analysis showed a statistically significant increase in TPRI during ICU hospitalization among non-survivors compared to stable levels among survivors (*p* < 0.01). Furthermore, CPI significantly declined among non-survivors during ICU hospitalization compared to stable levels among survivors (*p* < 0.01). Finally, the TBW among non-survivors increased during ICU hospitalization compared to stable levels among survivors (*p* < 0.01). 

### 3.3. Secondary Analysis

**Figure 1:**
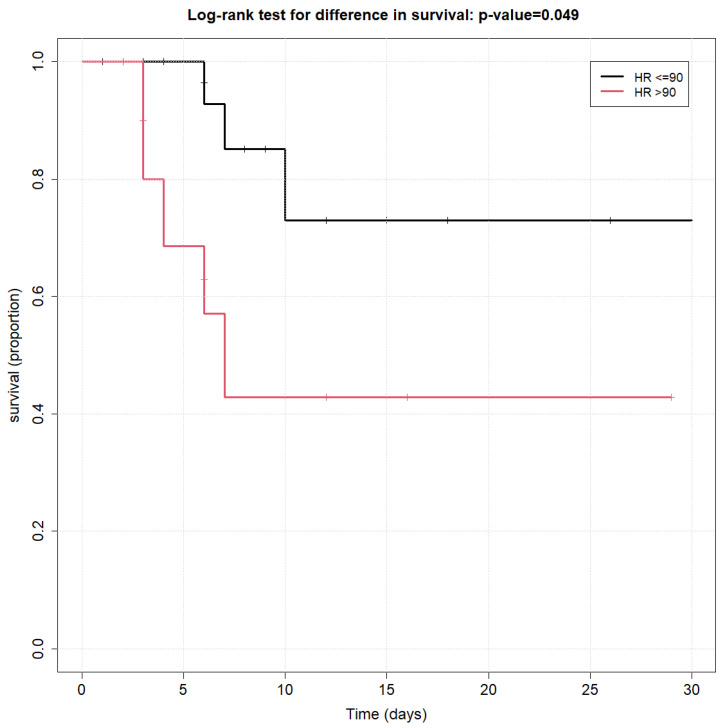
Kaplan–Meier survival curves of heart rate at admission to ICU.

Among all initial hemodynamic measurements tested (as listed above), the only variable that was found to be significantly associated with overall survival using the log-rank survival curve was an HR ≤ 90 at ICU admission (*p* < 0.01), as shown in Figure 1. 

### 3.4. Exploratory Analysis

**Figure 2:**
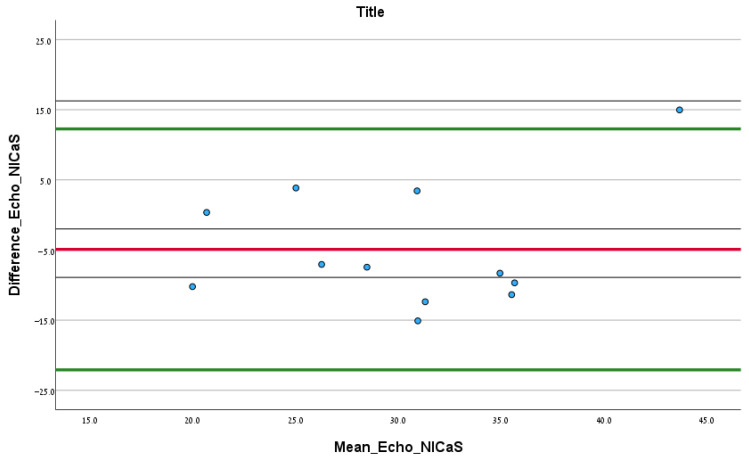
Bland–Altman analysis comparing the initial SI NICaS measurement and stroke volume index measured via echocardiography upon admission. The red line is the mean difference between the tests. The upper green line is the upper confidence interval and the lower green line is the lower confidence interval.

In total, 12.00 patients had documented echocardiography test results upon admission. According to the Bland–Altman analysis comparing SI via echocardiography and NICaS, the mean difference (SD) between the two tests was −4.91 (SD = 8.76) mL/m^2^, and the upper and lower confidence intervals were 12.26 and −22.08, respectively. No significant fixed error was found (difference of mean: −4.91, CI: −10.48–0.65, *p* 0.08), and no significant proportional error was found (correlation coefficient: 0.30, *p* 0.46), as shown in Figure 2.

## 4. Discussion

This study investigated the associated trends in the hemodynamic parameters obtained by the NICaS hemodynamic monitor and in-hospital all-cause mortality during COVID-19 ICU hospitalization. As future waves of similar viral illnesses are anticipated, and with the inevitability of additional mass illness of pandemic proportions, non-invasive hemodynamic monitoring may have a significant role to play. As mentioned earlier, there is a scarcity of studies that tracked advanced hemodynamic parameters throughout patients’ ICU stay through non-invasive means other than echocardiography. Based on a small sample size, we have demonstrated a good agreement between NICaS and echocardiography in measuring the stroke volume index, as depicted by the Bland–Altman analysis. This agreement may aid clinician’s confidence when relying on non-invasive measurements in a similar population of patients. The advantages of NICaS monitoring over echocardiography in the ease of use, the no-contact method, the ability to monitor several patients simultaneously, and the fast learning curve, prompt further consideration of the utility of non-invasive measurement in such patients and scenarios.

Indeed, using NICaS monitors in the ICU during COVID-19 was straightforward. Upon the decision to monitor the patient, the physician attached two stickers to opposing limbs; usually, the same stickers were efficient for about a week. The monitor was connected intermittently, and the data were transmitted via WIFI to a non-contagious environment, enabling the physicians to discuss the results and compare them to previous ones. 

Since our study was retrospective and the decision to monitor the patients with NICaS was at the physician’s discretion, we have compared these patients to a larger cohort of critically ill patients with COVID-19. The patients in our study had demographic characteristics and comorbidities similar to those of the larger cohort but had worse baseline prognostic scores. One possible explanation for this difference is the tendency to monitor the more critically ill patients. Another explanation is that NICaS was only used in designated ICUs, while the larger cohort consisted of a mixed population admitted to ICUs and intermediate units in the internal wards. This difference in the patient allocation may have created a selection bias. 

In our attempt to explore the correlation between hemodynamics and prognosis, we found CI, CPI, TBW, TPRI, and HR values related to survival and outcome. 

CI has been identified as a valuable marker for cardiac function and an essential component of adequate organ perfusion in the critically ill [11,12]. Our data are consistent with previous information, showing that CI would increase in patients who recover and decrease in patients who deteriorate. CPI is correlated with cardiac reserve, which is a direct indicator of overall cardiac function. It is a predictor of prognosis both in chronic and acute heart failure [13]. Our results support this correlation and show that COVID-19 patients with reduced cardiac reserve have a worse prognosis than those with good cardiac reserve.

Fluid management is a mainstay of critical care. The need for fluid resuscitation must be balanced against the consequences of fluid overload and subsequent organ dysfunction [14]. Prior research has demonstrated increased length of stay, duration of mechanical ventilation, and mortality in patients with a positive fluid balance [15]. Our study observed a significant increase in TBW in non-survivors compared to survivors. Although it may be challenging to differentiate between the effects of the natural course of the disease and the consequences of therapies administered during the ICU hospitalization, it is reasonable to assume that the increase in body water content is due to fluid resuscitation, fluid retention, and capillary leak. The measurement of TBW could be used not only to monitor volemic status and guide treatment but also to serve as a marker for severe disease and poor outcomes [16].

The increase in TPRI in non-survivors is more challenging to explain. One may assume that non-survivors cannot maintain adequate peripheral and systemic resistance. As COVID-19 has been known to cause micro and macroangiopathy, the disease could also contribute to decreased or increased local or global vessel resistance [17]. The exact effect may not be easy to calculate or predict. Changes in TPRI could also be representative of fluid shifts [18]. Due to the retrospective nature of our study, the increasing TPRI in non-survivors may also be due to higher vasopressor requirements. 

Sepsis may cause an adrenergic storm that may be associated with cardiac dysfunction. Tachycardia may also impair the oxygen supply–demand and cause cardiac injury. [19]. In our study, we have found tachycardia to be negatively associated with survival. Although HR on admission may be influenced by other factors like stress, fever, or measurement errors, it proved to be a marker of survival, most likely due to one of the two mechanisms mentioned. 

In an attempt to integrate these hemodynamic variables into a single description of the deceased patient, it is reasonable to assume that the combination of a decreasing CI and CPI, on the one hand, and an increasing TPRI and TBW, on the other hand, in the non-survivors suggests a COVID-19-induced sepsis cardiac injury. The cardiogenic shock is then followed by water retention and exacerbated by iatrogenic fluid administration. In an attempt to maintain perfusion pressure, vasopressor therapy results in an increase in after load, exacerbating stress on failing hearts. 

Interleukin (IL) 6 is a proinflammatory cytokine known to be involved in the pathogenesis of severe illness due to COVID-19 [20]. The use of monoclonal antibodies targeting IL-6 receptors has been recommended as part of multimodal treatment for severely ill COVID-19 patients. Despite evidence demonstrating the benefit of anti-IL6 treatment in severely ill patients and noting that CPI may be representative of the cardiac reserve, the relationship between CPI and death in patients undergoing anti-IL-6 treatment in this study most probably reflects the more severe course of disease in patients who received anti-IL-6 as a treatment.

In conclusion, non-invasive hemodynamic monitoring via NICaS is an effective method for evaluating patients with COVID-19 and may help guide clinical management via remote monitoring. In particular, it may help demonstrate the response of vasopressors, describe the hemodynamic and volemic status, and hint towards outcome. NICaS may be a reasonable alternative to echocardiography for specific values such as SVI, CI, TPRI (as an SVR equivalent), and volume status in this patient population. However, it does not provide data on pulmonary edema and permeability, as do lung ultrasound and minimally invasive monitoring using thermodilution techniques. NICaS enables advanced monitoring in cases where invasive monitoring is not feasible or entails increased personal risk due to hazardous infections. It allows adequate remote oversight of patients without exposing personnel to repeated measurements. Our study is non-interventional; hence, drawing clinical recommendations from our results is difficult. However, one may assume the decline in CI and fluid accumulation suggests a possible capillary leak and cardiogenic damage with resultant edema and the effects of fluid resuscitation. This may hint that a more restrictive fluid administration approach and a larger dependency on catecholamines support may be beneficial [21]. The significant negative correlation between tachycardia and prognosis emphasizes the importance of maintaining an adequate oxygen supply–demand ratio in these patients. Specifically in COVID-19 or any infectious pandemic resembling COVID-19 in terms of exposure risk and resources, cheap, non-invasive hemodynamic monitoring allows real-time decision making and improved therapy. The ease of use and the ability to monitor many patients simultaneously may help clinicians overcome the overflow of critically ill patients and provide appropriate personal therapy. When dealing with a new disease of unknown hemodynamic influence, extensive, real-time monitoring enables clinicians to gather information quickly and prepare accordingly. The use of remote advanced non-invasive hemodynamic monitoring may be cost-effective in lieu of other invasive procedures and interventions, as well as time- and resource-saving in a busy intensive care unit. 

## 5. Limitations

There are several limitations to this study. The analysis is observational and retrospective, and the sample size is small; hence, we did not aim to prove an improvement in prognosis or a reduction in mortality. The small sample size may not accurately represent the critically ill COVID-19 population and cannot compensate for a possible selection bias. The study group comprises 6% of critically ill patients at the time, but it does not reflect the cohort accurately since they represent the most severely affected patients. Moreover, several calculations (specifically CKD and DM) consist of even smaller sample sizes. Although we did not want to ignore this information, the results should be interpreted cautiously. The total sample size of the study patients and sample size of those who underwent echocardiography are small and may not necessarily represent the general population. Non-invasive hemodynamic data were obtained once every 24–72 h, not on a more consistent timeline. Some clinicians used real-time data for decision making, while others considered the data too experimental upon which to base therapies. This difference may have affected outcomes. 

## 6. Future Research

This research adds to the literature on the utility of non-invasive hemodynamic monitoring of the critically ill, specifically in COVID-19 patients, as well as common hemodynamic findings. Future research may include comparing and contrasting the hemodynamic profiles of critically ill patients between different waves and mutations of the endemic and pandemic viral diseases and evaluating the use of non-invasive cardiac output monitoring on morbidity and mortality in prospective, randomized, controlled trials.

More research may be directed to implementing artificial intelligence (AI) and machine learning in interpreting NICaS or other hemodynamic monitors [22,23]. For example, AI may be used to locate patients at risk of deterioration based on the hemodynamic parameters shown to predict worse prognoses like CI or TBW. 

## Figures and Tables

**Table 1:** Patient’s baseline characteristics and ICU admission variables according to in-hospital mortality.

		Total Patients (*n* = 29)	Patients Who Survived Index Admission (*n* = 20)	Patients Who Died during Index Admission (*n* = 9)	*p*-Value
Age in years, median (IQR)		66.00 (46.00–79.00)	56.50 (42.25–71.75)	81.00 (63.00–88.00)	0.03
Gender male, *n* (%)		21.00 (72.41)	13.00 (65.00)	8.00 (88.90)	0.37
BMI (kg/m^2^)		26.40 (23.97–30.88)	27.38 (24.44–32.25)	25.71 (23.18–28.30)	0.22
Pre-existing comorbidities					
	Cardiac disease *n* (%)	10.00 (34.50)	4.00 (20.00)	6.00 (66.70)	0.03
	Respiratory disease *n* (%)	7.00 (24.10)	5.00 (25.00)	2.00 (22.20)	1.00
	Hypertension *n* (%)	17.00 (58.60)	10.00 (50.00)	7.00 (77.80)	0.23
	Hyperlipidemia *n* (%)	13.00 (44.80)	9.00 (45.00)	4.00 (44.40)	1.00
	CKD *n* (%)	7.00 (24.10)	2.00 (10.00)	5.00 (55.60)	0.02
	DM *n* (%)	7.00 (24.10)	5.00 (25.00)	2.00 (22.00)	1.00
HR at admission, median (IQR)		94.00 (81.00–114.50)	93.50 (80–113)	102.00 (86.00–116.00)	0.39
Room air % saturation at admission, median (IQR)		89.00 (85.50–93.50)	89.00 (86.25–93.75)	89.00 (85.00–92.50)	0.60
Mews at admission, median (IQR)		7 (4.75–11.25)	6.00 (4.00–8.00)	14.00 (8.00–16.00)	*p* < 0.01
SOFA at admission, median (IQR)		2.00 (1.00–4.00)	2.00 (0.25–2.75)	5.00 (2.50–8.50)	*p* < 0.01
In-hospital length of stay in days, median (IQR)		12.00 (8.00–18.00)	13.50 (9.00–18.00)	7.00 (5.00–16.500)	0.10
ICU length of stay in days, median (IQR)		7.00 (3.50–12.00)	9.00 (3.25–14.25)	6.00 (3.50–8.50)	0.47

## Data Availability

Dataset available on request from the authors.
